# Supplementary material for: Remembering the Past with Today’s Technology: A Scoping Review of Reminiscence-Based Digital Storytelling with Older Adults
Source: Behav Sci (Basel). 2023 Dec 4;13(12):998. doi: 10.3390/bs13120998 (PMC10741197; doi:10.3390/bs13120998)
Supplement: Supplementary file 1 [file behavsci-13-00998-s001.zip › behavsci-2652183-supplementary.pdf]

Supplemental Table S1. Audit trial of this scoping review.

Stage of the knowledge synthesis	Main Elements
**Searching references**	Electronic databases searched: see Figure 1<br>Journals and previous reviews hand-searched:<br>• Journals: RBP Psychiatry; Aging and Mental Health; Hong Kong Journal of Occupational Therapy; Current Topics in Research<br>• Previous reviews: Elfrink et al., 2018; Huang et al., 2015; Park et al., 2019; Rios Rincon et al., 2021; Woods et al., 2018; Yen & Lin, 2018<br>Date of literature search in each database:<br>• AgeLine: 11/02/2021<br>• APA PsycInfo: 11/02/2021<br>• CINAHL Complete: 11/02/2021<br>• ERIC: 11/02/2021<br>• MEDLINE: 11/02/2021<br>• Social Work Abstracts: 11/02/2021<br>• Web of Science: 11/02/2021<br>Keywords and MeSH terms used: see Table 1<br>Number of references found in each database, journals, previous reviews, and included articles: see notes under Figure 1<br>Authors that were contacted for additional references: none<br>Number of references left after the removal of duplicates: 673
**Screening stage**	Number of references left after the screening of titles and abstracts: 35<br>Number of references left after the screening of the full-text assessment stage: 10<br>Detailed reasons of exclusion for each reference at the full-text assessment stage: see Figure 1
**Data extraction stage**	Data extraction files: see Table 5<br>Authors that were contacted for data validation or regarding missing data (as well as who responded): none
**Data synthesis stage**	Number of articles included in the different syntheses conducted (e.g., qualitative synthesis quantitative synthesis): 4 quantitative, 4 qualitative, and 2 mixed methods
